# Supplementary material for: Construction of a predictive model for bone metastasis from first primary lung adenocarcinoma within 3 cm based on machine learning algorithm: a retrospective study
Source: PeerJ. 2024 Mar 14;12:e17098. doi: 10.7717/peerj.17098 (PMC10944632; doi:10.7717/peerj.17098)
Supplement: Supplemental Information 2 [file peerj-12-17098-s002.docx]

The hyperparameters of our model are as follows:

{'objective': 'binary:logistic', 'base_score': 0.5, 'booster': 'gbtree', 'colsample_bylevel': 1, 'colsample_bynode': 1, 'colsample_bytree': 1, 'gamma': 0, 'gpu_id': -1, 'importance_type': 'gain', 'interaction_constraints': '', 'learning_rate': 0.3, 'max_delta_step': 0, 'max_depth': 8, 'min_child_weight': 2, 'missing': nan, 'monotone_constraints': (), 'n_estimators': 20, 'n_jobs': 0, 'num_parallel_tree': 1, 'random_state': 0, 'reg_alpha': 0, 'reg_lambda': 0.5, 'scale_pos_weight': 1, 'subsample': 1, 'tree_method': 'exact', 'validate_parameters': 1, 'verbosity': 0}
