# Supplementary material for: Construction of a predictive model for bone metastasis from first primary lung adenocarcinoma within 3 cm based on machine learning algorithm: a retrospective study
Source: PeerJ. 2024 Mar 14;12:e17098. doi: 10.7717/peerj.17098 (PMC10944632; doi:10.7717/peerj.17098)
Supplement: Supplemental Information 3 [file peerj-12-17098-s003.docx]

| Characteristics | SEER | Feicheng | P value |
| --- | --- | --- | --- |
| n | 19454 | 125 |  |
| Age, mean ± sd | 67.53 ± 10.206 | 66.408 ± 8.0873 | 0.125 |
| Sex, n (%) |  |  | 0.180 |
| female | 11425 (58.4%) | 66 (0.3%) |  |
| male | 8029 (41%) | 59 (0.3%) |  |
| Grade, n (%) |  |  | 0.007 |
| Grade II | 8698 (44.4%) | 62 (0.3%) |  |
| Grade I | 4648 (23.7%) | 35 (0.2%) |  |
| Grade III | 5990 (30.6%) | 25 (0.1%) |  |
| Grade IV | 118 (0.6%) | 3 (0%) |  |
| Race, n (%) |  |  | < 0.001 |
| White | 17676 (90.3%) | 0 (0%) |  |
| No white | 1778 (9.1%) | 125 (0.6%) |  |
| Marital, n (%) |  |  | 0.002 |
| yes | 10921 (55.8%) | 87 (0.4%) |  |
| no | 8533 (43.6%) | 38 (0.2%) |  |
| Tumor Size, mean ± sd | 19.963 ± 6.3636 | 20.712 ± 6.0479 | 0.189 |
| Tumor Site, n (%) |  |  | < 0.001 |
| Right upper | 7272 (37.1%) | 59 (0.3%) |  |
| right lower | 3352 (17.1%) | 0 (0%) |  |
| left upper | 4961 (25.3%) | 25 (0.1%) |  |
| right middle | 1117 (5.7%) | 20 (0.1%) |  |
| left lower | 2665 (13.6%) | 21 (0.1%) |  |
| Main bronchus | 87 (0.4%) | 0 (0%) |  |
| T Stage, n (%) |  |  | 0.491 |
| T1 | 13033 (66.6%) | 88 (0.4%) |  |
| T3 | 1805 (9.2%) | 8 (0%) |  |
| T2 | 3250 (16.6%) | 23 (0.1%) |  |
| T4 | 1366 (7%) | 6 (0%) |  |
| N Stage, n (%) |  |  | 0.030 |
| N0 | 13999 (71.5%) | 100 (0.5%) |  |
| N3 | 868 (4.4%) | 0 (0%) |  |
| N1 | 1501 (7.7%) | 5 (0%) |  |
| N2 | 3086 (15.8%) | 20 (0.1%) |  |
| Bone Met, n (%) |  |  | 0.008 |
| 0 | 18239 (93.2%) | 110 (0.6%) |  |
| 1 | 1215 (6.2%) | 15 (0.1%) |  |
